# Alveolar Organoids in Lung Disease Modeling

**DOI:** 10.3390/biom14010115

**Published:** 2024-01-16

**Authors:** Enkhee Purev, Karim Bahmed, Beata Kosmider

**Affiliations:** 1 Department of Microbiology, Immunology, and Inflammation, Temple University, Philadelphia, PA 19140, USA; 2 Center for Inflammation and Lung Research, Temple University, Philadelphia, PA 19140, USA; 3 Department of Thoracic Medicine and Surgery, Temple University, Philadelphia, PA 19140, USA; 4 Department of Cardiovascular Sciences, Temple University, Philadelphia, PA 19140, USA

**Keywords:** alveolar organoids, AT2 cells, lung, diseases, regeneration

## Abstract

Lung organoids display a tissue-specific functional phenomenon and mimic the features of the original organ. They can reflect the properties of the cells, such as morphology, polarity, proliferation rate, gene expression, and genomic profile. Alveolar type 2 (AT2) cells have a stem cell potential in the adult lung. They produce and secrete pulmonary surfactant and proliferate to restore the epithelium after damage. Therefore, AT2 cells are used to generate alveolar organoids and can recapitulate distal lung structures. Also, AT2 cells in human-induced pluripotent stem cell (iPSC)-derived alveolospheres express surfactant proteins and other factors, indicating their application as suitable models for studying cell–cell interactions. Recently, they have been utilized to define mechanisms of disease development, such as COVID-19, lung cancer, idiopathic pulmonary fibrosis, and chronic obstructive pulmonary disease. In this review, we show lung organoid applications in various pulmonary diseases, drug screening, and personalized medicine. In addition, stem cell-based therapeutics and approaches relevant to lung repair were highlighted. We also described the signaling pathways and epigenetic regulation of lung regeneration. It is critical to identify novel regulators of alveolar organoid generations to promote lung repair in pulmonary diseases.

## 1. Introduction

The lung is crucial for gas exchange between internal tissues and the external environment [1]. The alveolus is the lung’s functional gas exchange unit, which is covered by spherical sacs, with a diameter of 200–250 μm. In the lung, the alveolar epithelium regeneration after the damage is triggered by alveolar type 2 (AT2) epithelial cells [2]. They can self-renew and differentiate into very large, thin alveolar type 1 (AT1) cells participating in the gas exchange. Various animal models have been utilized in studies of the lung in health and disease. In two-dimensional (2D) culture conditions, the lung cells generate an epithelial monolayer and exhibit progenitor-like expression patterns [3]. However, considering the high complexity of lung tissue, 2D cell culture techniques have shortcomings due to limited approaches to studying communication between the cell matrix, neighbor cells, and biological function [4,5]. Lung organotypic models, 2D air–liquid interface (ALI), and three-dimensional (3D) organoid cultures have been considered platforms to study lung stem/progenitor cell differentiation and repair [6]. Thus, 2D ALI cultures can provide well-differentiated monocultures of human bronchial epithelial (HBE) cells [7]. The extracellular matrix (ECM) is one of the components of the cellular microenvironment [8]. Components of the ECM are critical determinants of cell function. HBE cells were seeded on inserts coated with collagen-I in 2D ALI cultures [7]. The main advantage of this model is the air interface, which promotes cell differentiation. It was compared with the 3D airway organ tissue-equivalent system, which showed a large amount of transcriptomic similarities with the 2D ALI culture. The reconstruction of the co-culture in vitro provides information on essential factors for tissue replacement, such as paracrine signaling and gap junctions [9]. Moreover, this model reflects in vivo characteristics. Among the various methods, the transwell co-culture system has been widely used. It was reported that 2D and co-culture using three different cell lines, A549 (lung epithelium), haCaT (keratinocytes), and HT-29 (intestinal epithelium) in the upper chamber and THP-1 (peripheral blood monocytes) in the lower chamber, can be used to study the toxicity of various compounds.

Multicellular spheroid and organoid systems are considered for studies of different cell types originating from various organs, including the lung, to improve the predictability of pre-clinical in vitro models [10]. Organoids created in a 3D system display tissue-specific functional phenomena and mimic features of the original organ. Nasal, bronchial basal stem cell-derived, and alveolar organoids were generated using human epithelial cells [11,12,13].

Persistent or dysregulated lung inflammation is a distinct feature of chronic respiratory diseases, such as severe asthma, chronic obstructive pulmonary disease (COPD), cystic fibrosis (CF), and bronchiectasis [14]. COPD is a chronic inflammatory respiratory disease of high global morbidity and mortality [15]. It is characterized by airflow limitation and heterogeneous endophenotypes. COPD is caused by chronic exposure to environmental factors, such as particles, gases, dust, and cigarette smoke [16]. Additionally, various factors can contribute to the development of this disease.

CF is a rare, monogenic disease evolved by mutations from the cystic fibrosis transmembrane conductance regulator *CFTR* gene [17]. A defective CFTR protein in the CF patients’ lungs leads to dehydrated surface liquid and compromised mucociliary clearance [18].

Lung cancer is a major cause of cancer mortality worldwide [19]. It is classified into three main types: adenocarcinoma, squamous cell carcinoma, and small-cell carcinoma. Additionally, several less frequent types were identified, including adenosquamous and large-cell neuroendocrine carcinoma. Non-small cell lung cancer (NSCLC) is the number one cause of cancer deaths in the USA [20]. A squamous subtype of NSCLC is considered the second most prevalent subtype of lung cancer, with a 20.2% survival rate [21].

Interstitial lung diseases (ILD) include parenchymal lung diseases with similar clinical properties and impaired repair after injury [22]. They possess divergent physiological mechanisms and are classified based on various phenotypes, such as autoimmune and unclassifiable ILD, exposure-related, sarcoidosis, idiopathic nonspecific interstitial pneumonia, and chronic hypersensitivity [22,23]. Idiopathic pulmonary fibrosis (IPF) belongs to ILD, and the disease’s main etiology is not well understood. It is a fatal lung disease characterized by intensive dyspnoea and loss of lung function [24]. IPF shows a diffused parenchymal phenotype, and AT2 cells are lost in the alveoli [25].

Acute respiratory distress syndrome (ARDS) is a highly morbid cause of acute hypoxemic respiratory failure characterized by inflammatory lung injury, in situ pulmonary vascular thrombosis, and microcirculatory dysfunction [26]. About 10–20% of patients who develop severe infection with acute respiratory syndrome-related coronavirus-2 (SARS-CoV-2), the virus responsible for coronavirus disease 2019 (COVID-19), develop ARDS. Approximately 90% of patients with COVID-19 in the intensive care unit developed ARDS, which led to high mortality rates [27]. Annually, it is estimated that there are 190,000 cases of ARDS in the USA, with hospital mortality of up to 40% [28].

Studies using isolated AT2 cells from the human lung and cultured AT2 and AT1 cells are limited [29]. Therefore, human-induced pluripotent stem cells (hiPSCs) can be used as an alternative approach to study alveolar epithelial dysfunction. Recently, the long-term organoid culture of the human distal lung containing AT2 and basal stem cells was used to model SARS-CoV-2 infection [30]. Single-cell RNA sequencing confirmed the expression of AT2 cell markers in the alveolar populations. Patient-derived xenograft (PDX) and organoid cultures have been considered useful pre-clinical tools to reduce the utilization of traditional 2D cultures [31]. Also, microfluidics can enhance the 3D cell cultures by boosting quality, functionality, throughput, real-time multi-index monitoring, and accurate simulation of the lung physiological microenvironment in vivo [32,33].

These models are the newest potential approaches for personalized medicine and drug screenings for lung diseases. In this review, we focus on recent studies of various lung 2D and organoid-based systems, improving our understanding of the pre-clinical platform, cell–cell interactions, and their further applications for personalized medicine, drug screenings, and future directions.

## 2. Lung Microenvironment

**Figure 1:**
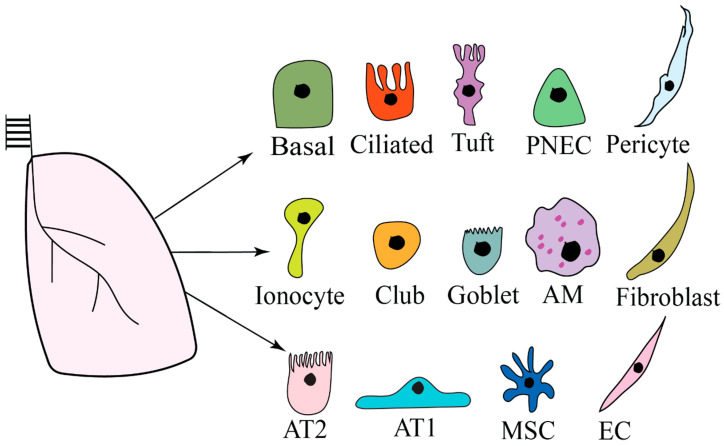
Lung microenvironment. Basal, ciliated, tuft, club, and goblet cells were identified. Pulmonary neuroendocrine cells (PNEC), pericytes, ionocytes, alveolar macrophages (AM), fibroblasts, mesenchymal stem cells (MSC), and endothelial cells (EC) are also present. The distal lung alveoli include alveolar type 2 (AT2) cells, which differentiate to alveolar type 1 (AT1) cells.

The lung has a complex microenvironment comprising various cell types subjected to different mechanical forces [34]. The human lung branches into 5 lobes and 18 bronchopulmonary segments, with 64,000 conducting airways [35]. The airway zone conducts air into the respiratory zone, where the gas exchange occurs. The airway epithelium is pseudostratified and consists of multiciliated cells, secretory goblet cells, and club cells scattered with neuroendocrine and basal cells (Figure 1) [36]. Multiciliated and secretory (including goblet and club) cell types originate from the tracheobronchial epithelial tissue-specific cells. They regenerate the pseudostratified airway epithelium and are located in the trachea and bronchi of the mouse and the upper respiratory tract of humans [37]. Neuroendocrine cells can be found as solitary cells [38]. Human neuroepithelial bodies are less stereotypically distributed throughout the airway epithelium. Cells2location mapping in Visium ST integrating spatial transcriptomes and single-cell RNA sequencing was used to identify the localization of ciliated basal epithelium, AT1, and AT2 cells [39].

Tuft cells are chemosensory cells spread in the lung’s upper and lower airways [40,41,42]. These cells located in the trachea are often called cholinergic brush cells and solitary chemosensory cells in the nasal respiratory mucosa. There are also chemosensory-like epithelial cells, named microvillar cells, in the olfactory mucosa. Tuft cells control innate and adaptive-phase eosinophilic lung inflammation [40]. They are a crucial source of cysteinyl leukotrienes (CysLTs), named for their canonical generation by leukocytes recruited or activated in inflammation. Moreover, calcium flux is important for generating CysLTs [43]. Also, rare cell-type ionocytes that express high levels of cystic fibrosis transmembrane conductance regulator CFTR in tracheobronchial regions were identified by single-cell RNA sequencing [44,45].

The adult human lung comprises about 480 million alveoli, mainly lined by AT2 and AT1 cells [29,46]. AT2 cells have a cuboidal phenotype and cover approximately 5% of the alveolar surface. They produce and secrete pulmonary surfactants, which reduce surface tension at the air–liquid interface and are required for lung function [46]. AT2 cells release surfactant proteins A, B, C, and D, lipids, cytokines/chemokines, and other molecules crucial for lung defense and homeostasis [29,47,48]. Lamellar bodies (LBs) in AT2 cells are lysosome-related organelles specializing in modifying surfactant components and storing pulmonary surfactants for secretion [49]. AT2 cells function as facultative stem cells to maintain homeostasis and lung damage repair via self-renewal and differentiation to AT1 cells [50,51]. AT2 cell differentiation to AT1 cells is regulated by the Notch, Hedgehog, and Wnt signaling pathways. In addition, the AT2 cell progenitor gains an AT2-to-AT1 transitional cell state expressing *KRT8*, *HBEGF*, or *AREG* genes [52]. Any deficiencies in surfactant protein impair normal lung functions [53].

The healthy adult lung contains two types of resident macrophages: a self-renewing cluster of embryonic-derived alveolar macrophages (AMs) and interstitial macrophages near the larger airways and in the lung interstitium, respectively [54]. Tissue-resident AMs occupy the alveolar lumen and are crucial for lung homeostasis and response to pathogens and pollutants [55].

In addition, there are also distinct types of stromal cells in the interstitial region, including mesenchymal stem cells (MSCs), pericytes, endothelial cells (ECs), and immune cells [56]. MSCs are multipotent progenitor cells that can differentiate into various cell types, migrate to injured lung parts, and contribute to lung regeneration [57]. For instance, one of the paracrine factors, prostaglandin E2 (PGE2), produced by MSCs modulates inflammatory and fibrotic disease. Pericytes are mesenchymal-originated mural cells that cover capillary networks in the circulatory system and directly connect with endothelial cells [58]. Pulmonary microvascular pericytes generate collagen to preserve vascular stability in pulmonary tissue [59]. The endothelium is a complex physical barrier between blood, air, and stromal tissue [60]. It is metabolically active and participates in the control of inflammation, leukocyte trafficking, gas and nutrient exchange, homeostasis, angiogenesis, vascular tone, and endocrine signaling [60,61]. The cellular diversity of the human lung endothelium has not been completely elucidated. Lung endothelial cells are a crucial source of IL-1β, a comprehensively studied member of the IL-1 family of proteins that are key regulators of inflammation and tissue homeostasis in the lung [62,63]. The lung microenvironment also contains fibroblasts [34]. They modulate the release of proinflammatory cytokines, such as IL-6, and chemokines from macrophages. Fibroblasts are a major component of the stromal cells and are active in ECM synthesis [64]. They are heterogeneous cells and are involved in fibrotic disease development [65]. Multiple lung fibroblast subtypes release matrix proteins and have different invasion, proliferation, and contraction capacities. However, lung tissue engineering has faced challenges in recapitulating in vitro human models using various materials that can mimic the native lung microenvironment [66]. New techniques such as single-cell transcriptome and immunoprofiling have been applied to profile human lung mast cells [67]. For instance, the molecular events in tumor cell–immunocyte interactions in the lung adenocarcinoma microenvironment need to be studied [68]. Moreover, determining drug-resistance genes could help in the prognosis of lung adenocarcinoma and improve the clinical efficacy of drugs [69]. Recently, the effects of mucus on airway macrophage activation and plasticity on gene and protein expressions and functional changes were determined [70]. This evaluation may direct us toward developing and improving immunomodulatory therapies.

## 3. Lung Organoids

In vitro cell cultures are harnessed to display the mechanisms of in vivo behaviors, including cell differentiation, migration, growth, and mechanics [4]. The advantages of 2D ALI cultures were associated with promoting differentiation and pseudostratification of HBE [7]. The ALI system allows mucus production and ciliation of the HBE and other important functional properties. The 2D planar microfluidic model is considered one of the main approaches reflecting air and liquid flow on the apical and basal sides of HBE. The 3D in vitro airway model with the formation of differentiated HBE cultures, cell–cell interactions, and ECM was reported. The ALI system is considered the gold standard in the respiratory field [71]. Although ALI culture represents in vivo like structures, it requires about 21 days of culture and is labor-intensive to maintain. Lung organoids may improve our understanding of in vitro and in vivo systems. Organoids are self-resembling 3D structures that can be generated from stem cells, tissue-specific progenitor cells, iPSC, and mass cultures usually embedded in the Matrigel with nutrients [5,72,73]. In addition, they serve as an effective tool for drug screening, disease modeling, and regenerative medicine [74]. One of the first 3D lung cell cultures, called organoids, was established using a digested fetal tissue, in which cell suspension included epithelial, mesenchymal, endothelial, and hematopoietic cells [5]. They consisted of about 80% bronchioalveolar organoids in an initial culture, and the remaining populations had alveolar and bronchiolar phenotypes [73]. Human lung organoids have been created to study lung tissue development, disease pathogenesis, and treatment [75]. Lung organoids include alveolar, bronchoalveolar, iPSC-derived, and airway cultures (Table 1) [72,76,77].

Thus, 3D primary human airway epithelial cultures were utilized as an in vitro model to study the function of different factors [84]. Various methods are used to access an apical membrane in airway organoid cultures [72]. Apical-out airway organoids were applied in a high-throughput assay for the antiviral drug screenings. In this platform, 3D organoids were generated from the proximal airway epithelium to create a novel in vitro lung model for infection with common respiratory viruses, such as enterovirus D68, influenza A, influenza B, and rhinovirus A16, and utilize it for the antiviral drug screening assays. Lung organoids were generated using two systems, ALI culture on inserts exposed to air, and submerged culture without air exposure [5]. Complex and more diverse organoids were created under former conditions. The submerged system formed a cystic phenotype, and these cultures were not investigated further due to morphology. Primary human airway epithelial cells cultured under ALI have advantages, such as reflecting lung tissue’s architecture and cellular complexity [85]. In this study, the effects of SARS-CoV-2 inhibitors were analyzed using human airway epithelial cells under ALI conditions.

Lung organoids are suitable in vitro models to study AT2 cell function. It has been reported that iPSCs differentiation to the monolayered epithelial iAT2 spheres (alveolospheres) can be used to analyze an AT2-exclusive disease-associated variant (SFTPC*^I73T^*) [86]. This model provides a pre-clinical platform to define the impact of AT2 cell dysfunction in ILD, evaluate treatments, and guide toward personalized medicine.

COVID-19 is caused by the SARS-CoV-2 virus, which typically infects the lower respiratory tract, inducing inflammation and diffuse alveolar damage [78,87]. To recapitulate this disease scenario, a human pre-clinical COVID-19 lung model was developed using an alveolosphere system to mimic AT2 cell infection with SARS-CoV-2 cells in vitro. Self-renewing AT2 cells express HTII-280, SP-C markers, and the SARS-CoV-2 entry receptor, angiotensin-converting enzyme 2 (ACE2), in alveolospheres. Additionally, infected AT2 cells displayed proinflammatory transcripts related to viral infections, including type 1 and type 3 interferon genes and their downstream targets. Moreover, alveolar cultures were treated with hydroxychloroquine and remdesivir. The latter was a strong inhibitor of SARS-CoV-2 replication in alveolar cultures, showing a 9-log reduction in viral N gene expression compared to untreated controls.

However, the limitations of lung organoids include the absence of endothelial cells and immune cell co-culture conditions, naïve ECM components, and physiological-like mechanical stress [88,89,90]. Spheroidal models address some limitations of 2D monoculture models; however, there is a lack of an external ALI for cilia and mucus analysis [7]. In addition, the success rate of growth and purity of tumor organoids vary [91]. Also, ethical issues associated with human challenge models restrict their use and access [92]. Taken together, lung organoids can be considered an excellent potential platform for studying virus pathobiology, lung disease development, and robust screening tools for drug candidates in vitro.

## 4. Generation of Alveolar Organoids

The distal lung consists of terminal bronchioles and alveoli, enabling gas exchange [30]. We have previously shown that primary human AT2 cells can maintain their differentiated phenotype in a 2D system [93]. Matrigel and extracellular matrix hydrogels are abundant in type IV collagen and laminin, the main characteristics of the basement membrane [94]. It was reported that alveolar organoids derived from hiPSC can be embedded in type IV collagen and laminin. Alveolar organoids were successfully emulated by co-culture of AT2 cells with PDGFRα^+^ fibroblast populations from the alveolar stem niche or lung endothelial cells [95].

Mice are often preferred for pre-clinical studies [96]. Additional models are generated to extrapolate experimental outcomes observed in vivo to humans. A human immunosystem mouse model was used to study the pathogenesis of SARS-CoV-2 variants. Human alveospheres provided additional advantages to studying infection with the SARS-CoV-2 virus, followed by transcriptomic analysis by global RNA sequencing [78]. Alveolar lung organoid co-culture is a ready-to-use system for studying the regulations of innate immune response and apoptotic genes in AT2 cells, AT2 cell differentiation to AT1 cells, and SARS-CoV-2 infections to screen respiratory antiviral drugs [78,95,97]. Macrophages were also applied in the co-culture to reflect a barrier between capillary blood and alveolar air [98]. The impact of different chemical substances based on their acute toxicities was evaluated. iPSC-derived alveolar epithelial cells (iAECs) and iAEC-based organoids and macrophage co-culture systems were utilized. Moreover, AT2 cell-derived alveolar organoids were developed without fibroblast using fibroblast-expressed ligands and small-molecule inhibitors [99]. Human AT2 cells were cultivated with a specific mixture of Matrigel containing Jagged-1 and culture medium with Fgf7, Noggin, SB431542, and CHIR99021 to establish fibroblast-free alveolar organoids.

In addition to successful applications of Matrigel for the alveolar organoid formation, chemical and physical properties of hydrogel were also used [100]. The hyaluronic acid hydrogel was customized to contain pre-defined microcavities to generate lung alveolospheres with uniform sizes in each microwell. In this model, AT2 cells in human iPSC-derived alveolospheres express surfactant proteins and characteristics in the Matrigel-free system.

In addition to developing alveolar models, promising approaches are being enhanced using isolated primary AT2 cells and PSCs, which can provide additional advantages for lung disease treatment options (Table 2).

## 5. Approaches for Lung Regeneration

Regenerative medicine includes transplantation, tissue engineering approaches, stem or progenitor cell therapy, or a combination of these elements [108]. Lung transplantation is a life-saving approach for end-stage lung diseases [109]. Annually, 2700 lung transplants are performed in the United States, with a 1-year median survival of over 90% and a 3-year survival of over 75%. The survival rate after lung transplant remains poor and has not significantly improved over the past several decades [110]. Standard-of-care immunosuppressive approaches have failed to achieve acceptable long-term graft and patient survival. Although lung transplantation is a promising option for patients with end-stage respiratory disease, the difficulty of finding partially matched lung donors has led to an extensive search for alternative solutions [111]. For this reason, stem cell-based therapeutic strategies were investigated in IPF, considering their potential to repair damaged lungs and restore their function [112]. Also, additional in vitro, in vivo, pre-clinical, and clinical studies are required to improve our understanding of lung regeneration. Cell-based therapies have become promising for patients with lung disease and acute lung injury [113]. MSCs have been tested in many clinical trials. An intratracheal administration of MSCs labeled with quantum dots visually confirmed uniform cell delivery into human alveoli. There is considerable interest in investigating cell-based therapeutics to ameliorate lung injury or treat disease [114].

In addition, telocytes (TCs) are an emerging type of interstitial cells with regenerative capacity [115,116]. TCs were found in the interstitial space of terminal bronchioles and demonstrated that their telopodes connected with alveolar epithelial cells and other cells in lung tissue. It has also been reported that TCs located in the interstitial space between the smooth muscle fibers, bronchiolar tree stroma, bronchiolar epithelium basement membrane, and neuroepithelial bodies are in contact with fibroblasts, dendritic reticular cells, lymphocytes, or stem cells [115]. They are also present in the walls of blood vessels and capillaries, suggesting they may be involved in the air–blood barrier. TCs form networks through homo- and heterocellular interactions, indicating their role in intercellular signaling in lung development. Moreover, TCs have a distinguishable ability to convey genetic materials and signaling molecules to stimulate stem and immune cells by releasing extracellular vesicles [116]. During the regeneration of injured tissue, TCs play a role as progenitor and nutrient cells, including angiogenesis, through the secretion of vascular endothelial growth factor (VEGF). Consequently, TCs may support lung injury repair [115].

Human micro-physiological systems, such as organ-on-a-chip microfluidic devices, have been developed to culture cells in organ-specific 3D environments to recapitulate the complex biological, structural, and physical states in vivo [117]. The alveolus chip, designed for physical breathing motions to recapitulate the human alveolar–capillary interface, cell–ECM interactions, and epithelial–endothelial crosstalk and vascular fluid flow, can be applied to study lung regeneration. Studies have shown that the alveolus chip can mimic human pulmonary disease [118]. Adenoviral vector-mediated CD98 HH domain gene delivery in alveolus-on-a-chip protected against pulmonary vascular leakage and could lead to additional potential therapeutics for pulmonary edema. Many scaffolds, such as synthetic and natural materials, have been applied to study regeneration in vitro [119]. A collagen scaffold is often used, and it is characterized by low antigenicity, favorable biocompatibility, biodegradability, excellent mechanical stability, and structural guidance for cell growth compared to other materials.

Collagen, the main structural protein of extracellular tissues, has been utilized as a scaffold to treat injuries [120]. The high-affinity leptin receptor (LEPR)-binding peptide in the collagen scaffold hugely enhanced endogenous mesenchymal cell recruitment and regeneration of damaged lung tissue. Glycosaminoglycans are the main component of the ECM and could contribute to the recruitment of immune and vascular cells, inducing potent and localized growth factors to initiate tissue regeneration [121]. Numerous molecules, such as collagen, gelatin, and alginate, have been used in tissue engineering to mimic native ECM scaffolds.

Lung development involves specialized bronchiolar and alveolar epithelial cells [122]. Inherited deficiency of *SFTPB* is a rare genetic syndrome of lethal respiratory phenomenon in full-term newborn infants. This syndrome is most commonly caused by homozygous, frameshift, loss-of-function mutations in the *SFTPB* gene (p.Pro133GlnfsTer95, previously known as 121ins2), allowing it to be a good target for gene therapy, which can be used successfully to repair or inactivate mutations in human in vitro cells. There have been successful attempts to gene edit *SFTPB* deficiency using nuclease-encoding mRNA, electroporation-mediated gene delivery, and CRISPR technologies. Highly efficient lentiviral infection of the wild-type *SFTPB* gene into the mutated hiPSC line showed successful transcription and translation of *SFTPB* in organoids.

*ABCA3* is required for the packaging and secretion of surfactant lipids and lung function at birth [46]. It is located on human chromosome 16p13.3 and encodes a 1704-amino acid (190-kDA), a multi-membrane-spanning protein expressed highly in AT2 cells. High loss of *Abca3* resulted in respiratory collapse and death caused by surfactant deficiency, alveolar-capillary leakage, and inflammation, consistent with the requirement of *ABCA3* for lung function in newborn infants. Non-lethal deletion of *ABCA3* caused lung injury and inflammation. However, a selective regeneration of *ABCA3*-sufficient AT2 progenitor cells was observed.

Moreover, Abelson kinases *Abl1* and *Abl2* are a family of nonreceptor tyrosine kinases that regulate many cellular events during lung development and homeostasis [123]. Abl kinases control diverse downstream targets, some of which regulate lung epithelial cell injuries. These include transcriptional co-activators of the Hippo (Yap1, Taz) and Wnt/β-catenin signaling pathways, which have been implicated in alveolar regeneration.

An additional protein, TRIM72, was found to be a critical component of the “repair kit” in alveolar epithelial cells [124]. It plays a role in the repair of alveolar cell membrane disruptions, and exogenous recombinant human TRIM72 protein (rhT72) demonstrated tissue-restoring properties. It was shown that *TRIM72* effectively protects from lung injury and has lower concentrations in healthy lung regions. Also, rhT72 reduced lactate dehydrogenase release in human primary alveolar epithelial cells.

Between 8% and 15% of familial IPF cases relate to mutations in telomerase or telomere-protective proteins [125]. Telomere dysfunction in AT2 cells may contribute to pulmonary fibrosis [126]. Human iPSCs were used to generate dyskeratosis congenita (DC) mutant iAT2 cells with shortened telomeres to interrogate how telomere dysfunction can affect AT2 cell function. Shortened and uncapped telomeres were associated with a defect in alveolosphere formation using iAT2 cells. The GSK3 inhibitor, CHIR99021, which mimics the output of canonical Wnt signaling, enhanced telomerase activity and rescued the defects. Wnt agonists may serve as potential therapies for DC-related pathologies. Organoid modeling indicates that human respiratory airway secretory cells can act as progenitors for AT2 cells, which is crucial for regenerating the alveolar environment [127]. New insights provide novel therapeutic opportunities for repair in lung diseases. In addition, it is important to analyze lung developmental stages and utilize the obtained results in regenerative medicine [128]. Identifying the function of various cell types in the lung could advance transplantation technologies and therapies [129].

## 6. Signaling Pathways in Lung Regeneration

**Figure 2:**
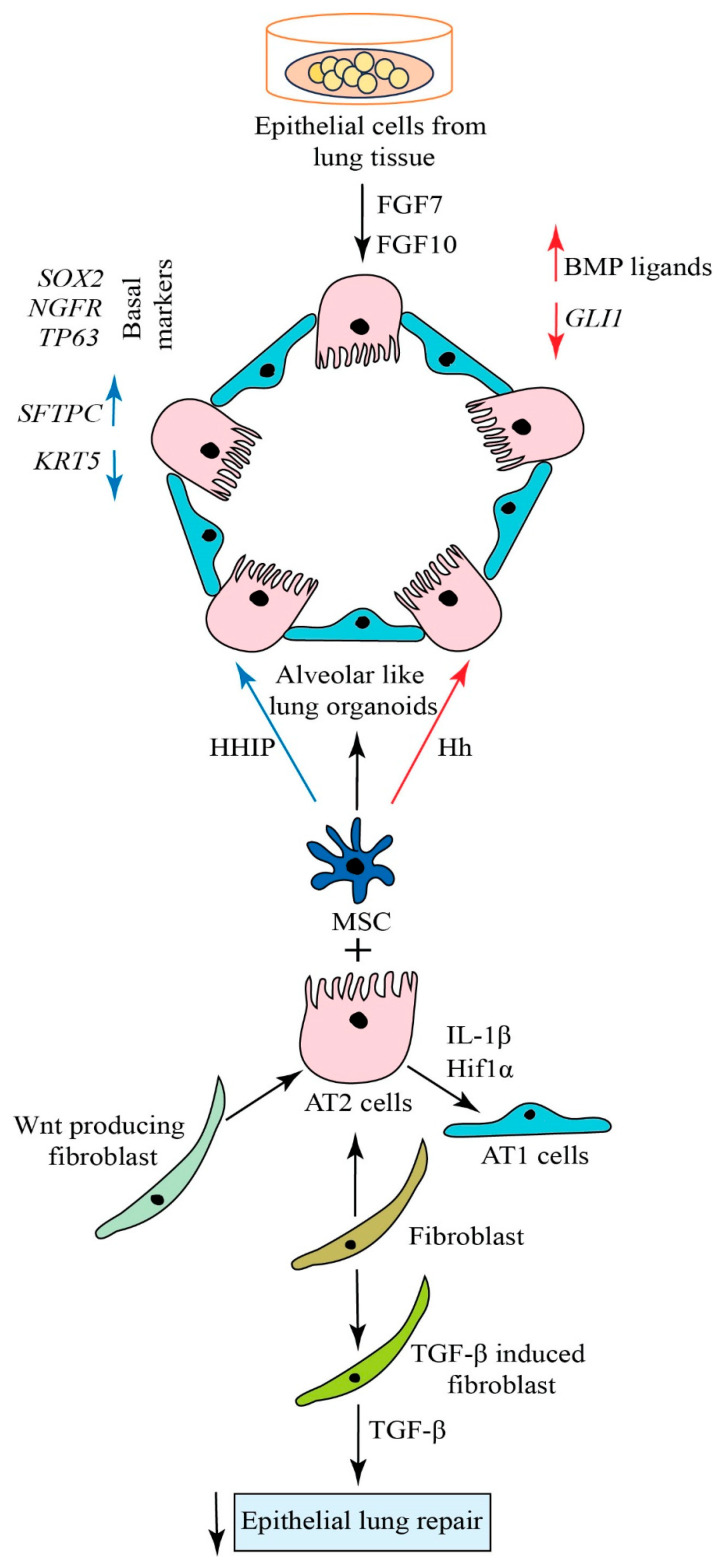
A schematic illustration of signaling pathways in lung regeneration. An induction of distal-like organoids using effective FGF ligands. Wnt-producing fibroblasts contribute to regulating stem cell features of AT2 cells. Damage-associated transient progenitors induced by IL-1β-driven Hif1α participate in alveolar regeneration and AT1 differentiation. AT2 cells can transdifferentiate into Krt5^+^ basal cells when co-cultured with adult human lung mesenchyme cells. Supplementation with recombinant HHIP decreased *KRT5* and increased *SFTPC* expression. Hh transcription factor, *GLI1*, was attenuated and increased BMP ligands. In this organoid system, AT2-derived basal cells express basal markers such as *SOX2*, *NGFR*, and *TP63.* TGF-β-induced myofibroblast differentiation diminishes the lung epithelial repair.

Multiple signaling pathways, such as fibroblast growth factor (FGF), bone morphogenic protein, Sonic Hedgehog, epidermal growth factor (EGF), retinoic acid, and HIPPO pathways, are involved in the regulation of lung development and regeneration [130]. Within these signaling pathways, FGF2, FGF7, FGF9, and FGF10 induced lung organoid formation in a 3D matrix condition. Specifically, FGF7 and FGF10 had the highest capacity to promote lung organoid formation, branching, and differentiation towards distal lung lineage. In the distal lung, alveolar regenerations are turned on after injury via the proliferation and differentiation of AT2 cells to AT1 cells to facilitate gas exchange [131]. Mesenchymal cell subtypes, and Wnt-responding and -producing fibroblasts, contribute to regulating stem cell properties and features of AT2 cells. It was reported that inflammatory niches triggered by the IL-1β and Hif1α signaling pathways regulate regeneration and the differentiation of AT2-lineage cells (Figure 2).

The effects of various signaling pathways were studied in alveolar organoids. It has been shown that human AT2 cells can transdifferentiate into Krt5^+^ basal cells when co-cultured with lung mesenchymal cells in 3D organoids (Figure 2) [25]. To analyze AT2 cell differentiation, pathological niche factors were systematically evaluated in human AT2 cell-derived organoids. It is known that hedgehog antagonist HHIP was upregulated in MRC5 cells and found in human AT2 cells. Immunophenotyping revealed that human AT2 cells co-cultured with adult human lung mesenchyme (AHLM) showed intermediate and early basal cell markers KRT8 and KRT17, respectively, in ~65% of organoids. In this system, supplementation with recombinant HHIP decreased *KRT5* and increased *SFTPC* expression. In addition, the Hh transcription factor, *GLI1*, was attenuated, and BMP ligands increased. The analysis showed that human AT2-derived basal cells express canonical basal markers, such as *SOX2*, *NGFR*, and *TP63*. They overexpress markers previously reported upregulated in the IPF epithelium, such as *KRT14*, *VIM*, and *MMP7*. In addition, a histologic comparison of normal and IPF lungs and human AT2-derived organoids shows that these IPF biomarkers are specifically expressed in basal cells from IPF and human AT2-derived organoids but not normal lungs. TGF-β-induced myofibroblast differentiation impairs the ability of human lung fibroblasts to support epithelial repair [132].

Wnt/β-catenin signaling modulates progenitor cell fate during lung development and in various adult tissues [133]. This pathway was involved in the proliferation, motility, and maintenance of stem cells to, thereby, contribute to regeneration [134]. WNT ligands (19 in humans) are evolutionarily conserved secreted glycoproteins, indispensable for proper organ development, especially lung development [135]. Specific WNT ligands can activate the β-catenin-dependent (canonical) or -independent (noncanonical) pathways by acting on various transmembrane receptors. Expression and post-translationally modified WNT-5A transcript were reported to be enhanced in lung tissue in COPD patients. Studies have shown that the origin of myofibroblasts is still debatable [136]. It is critical to determine potential factors that convert fibroblasts to myofibroblasts [137]. In addition to signaling molecules, a number of cytokines are responsible for alveolar regeneration [138]. Also, a potential therapeutic target, an activated leukocyte cell-adhesion molecule for IPF, may aid in the development of novel strategies for managing treatment directions [139]. Understanding the defects between fibroblast–epithelial interactions can lead to developing promising therapeutic targets for correcting epithelial repair in chronic lung diseases.

## 7. Challenges and Future Directions

**Figure 3:**
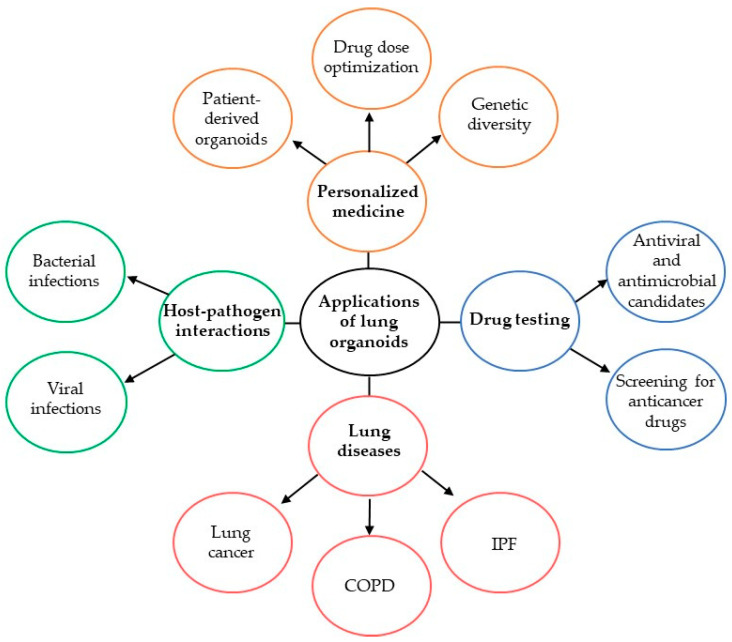
Applications of lung organoids. Lung organoids can be utilized to study anti-cancer, antiviral, and antimicrobial drugs. Personalized medicine provides a unique value in the applications of lung organoids. They have been used to model lung diseases such as lung cancer, COPD, and IPF. Also, lung organoids are applied to study host-pathogen interactions.

In the last few decades, studies of lung organoids have been expanded. They have been established from the various resources, as reviewed above. One of them is hiPSCs, which are considered for personalized drug screening, differentiating lung organoids, cell–cell interaction studies, respiratory viral models, and lung disease research [74,140,141,142]. The characterization and optimization of airway lung organoids from hiPSCs may be expanded and utilized for various further applications. Currently, different types of lung organoids may be used in cell-based therapies, host–pathogen interactions, recapitulation of lung diseases, and drug testing (Figure 3). However, challenges in efficiently generating pure lung cancer organoids could be a main issue in addition to the efficiency of selection strategies to remove unwanted populations and selection based on culture media formulations [91]. In addition, freezing protocols for airway organoids are not as well optimized as the basal cells [74,143]. The therapeutic potential of cell-based therapy for lung disease, determining cell seeding and doses, needs to be improved [113]. Lung organoid models may be applied for studies of respiratory virus infections [144]. Moreover, they are beneficial to determine a host immune system in mice and humans. Lung organoids and multiple-tissue organ-on-a-chip platforms could be applied to study the pathological processes, enhance lung epithelial repair, and treat pulmonary abnormalities [145]. Further, the 3D lung-on-chip model may be considered an anatomically inspired membrane-based organ-on-a-chip model of epithelial and endothelial tissue barriers, such as bronchioles, renal tubules, intestinal villi, blood or lymph vessels, in the future.

## 8. Conclusions

This review focuses on alveolar organoids and approaches to lung regeneration. We provided information regarding their generations from the lung’s airways, bronchoalveolar, and alveolar regions. Organoids have been utilized effectively in modeling human diseases, studying host–pathogen interactions, and drug screening. Patient-derived lung cancer organoids have been considered alternative in vitro models for anti-cancer therapeutics screening and establishing a biobank for individual patients. For personalized medicine for lung cancer, in vitro tissue culture or tumor spheroid culture in a 3D condition has been utilized to predict the anti-cancer treatment and original tumor behavior. Han et al. utilized cisplatin-resistant patient-derived organoids (PDOs) to determine the functions of solamargine [146]. It was shown to inhibit the cell growth of PDO and the colony formation of lung cancer cell lines NCI-H1299 and NCI-H460. Furthermore, solamargine increased the percentage of cells in the G0/G1 phase.

Studies using patient-derived alveolar organoids may provide a potential platform for personalized therapy, facilitate drug discovery trials, and enable long-term biobanking of cells (Figure 3). Improving our knowledge of alveolar organoid growth is critical to promoting lung regeneration and utilizing it for pulmonary disease treatments. The development of organ-on-a-chip models that recreate the tissue architecture is biologically relevant. It permits integrating the dynamic biomechanical changes and cellular interactions in tissues to facilitate pre-clinical human in vitro models [147].

Moreover, microfluidic lung tissue on high-throughput assay platforms may be used to develop respiratory viral infection and disease models for drug discovery [148]. This study focused on cell population characterization by fluorescence microscopy and flow cytometry using vascularized alveolar and bronchiolar multi-chip models. To this end, alveolar organoids may provide a potential platform for personalized therapy, facilitate drug discovery trials, and enable long-term biobanking of cells [78,149,150].

## Figures and Tables

**Table 1:** Types of human lung organoids.

Lung Organoids	Cell Types	Applications	References
AT2 cell-derived organoids iPSC-derived AT2 cells organoids	AT2 cells and MRC5 cells	SARS-CoV-2 infection in vitro model Organoid generations	[78,79,80]
Bronchial organoids	Normal bronchial epithelial cells	SARS-CoV-2 infection	[81]
Small airway and lung bud tip organoids	Small airway stem cells and fetal lung epithelial bud tips	SARS-CoV-2 infection	[82]
Tracheobronchial organoids	Tracheobronchial epithelial cells Cystic fibrosis bronchial epithelial cells	Functional studies	[83]

**Table 2:** Generation of lung organoids in disease modeling.

Lung Disease Origin	Type of Lung Organoids	References
IPF	hPSCs- derived alveolar organoids, iPSC-derived alveolar organoids	[101,102]
COPD	Nasopharyngeal and bronchial organoids Alveolar organoids	[15,103]
Lung cancer	Small cell carcinoma and adenocarcinoma Primary cancer tissues Patient-derived tumoroid	[104,105,106]
Cystic fibrosis	Tracheobronchial epithelial organoids Airway organoids from CF patients	[83,107]
